# Tumor necrosis factor-alpha and Fas/Fas ligand signaling pathways in chronic spontaneous urticaria

**DOI:** 10.1186/s13223-019-0332-7

**Published:** 2019-03-14

**Authors:** R. Grzanka, A. Damasiewicz-Bodzek, A. Kasperska-Zajac

**Affiliations:** 1 Clinical Department of Internal Diseases, Dermatology and Allergology, SMDZ in Zabrze, Medical University of Silesia in Katowice, ul. M. Curie-Skłodowskiej 10, 41-800 Zabrze, Poland; 2 Department of Chemistry, SMDZ in Zabrze, Medical University of Silesia in Katowice, Katowice, Poland; 3 European Center for Diagnosis and Treatment of Urticaria (GA2LEN UCARE), Zabrze, Poland

**Keywords:** Chronic spontaneous urticaria, Tumor necrosis factor alpha (TNF-α), Soluble tumor necrosis factor receptor type 1 (sTNF-R1), Soluble tumor necrosis factor receptor type 2 (sTNF-R2), Soluble Fas (sFas), Soluble Fas ligand (sFasL)

## Abstract

**Background:**

There is increasing evidence pointing to the important role of tumor necrosis factor-alpha (TNF-α), a key inflammatory and apoptotic mediator in urticarial inflammation. However, the role of the TNF-α system and Fas/Fas ligand (FasL) in the apoptosis-inducing pathways in chronic spontaneous urticaria (CSU), remain unclear.

**Aim:**

To determine circulating concentrations of TNF-α, soluble TNF-α receptor type 1 and type 2 (sTNF-R1 and sTNF-R2, respectively) as well as soluble Fas (sFas) and FasL (sFasL) in CSU subjects.

**Methods:**

Serum TNF-α, sTNF-R1, sTNF-R2, sFas, sFasL concentrations were measured using enzyme-linked immunosorbent assay in CSU subjects and in the healthy subjects.

**Results:**

TNF-α concentrations were significantly higher in CSU subjects and moderate-to-severe CSU than in the controls, while there were no significant differences in TNF-α concentrations between subjects with mild CSU and the controls. sTNF-R1 and sTNF-R2 concentrations were significantly higher in all CSU and moderate-severe CSU subjects vs. the controls. Serum concentrations were also significantly higher in mild CSU vs. the controls, but not in moderate-severe CSU vs. mild CSU. No significant differences were observed in sFas and sFasL concentrations between CSU subjects and the healthy controls. Significant correlations were found between concentrations of TNF-α and its receptors, as well as sTNF-R1 and sTNF-R2, but not with the urticaria activity score (UAS). There was no relationship between TNF-α/sTNF-R1/sTNF-R2 and sFas/sFasL pathways in CSU.

**Conclusions:**

CSU is associated with the activation of the TNF-α/receptors signaling pathway, marked by increased circulating concentrations of TNF-α, sTNF-R1 and sTNF-R2, which are related to each other in this disease. In contrast, the circulating sFas/FasL system is not up-regulated in CSU, and sFas/sFasL may not be a useful marker of the activity/severity of urticarial processes. Considering the lack of significant changes in sFas/sFasL (mainly reflecting systemic apoptosis) in CSU patients, it appears that elevated serum TNF-α concentrations are related to its pro-inflammatory function rather than an enhanced systemic apoptotic response in CSU.

## Introduction

Chronic spontaneous urticaria (CSU) is associated with autoimmune processes and a systemic inflammatory response [1–3].

Tumor necrosis factor alpha (TNF-α) plays a central role in inflammatory processes, immune regulation and apoptosis. TNF-α exerts its action via cell-surface receptors. They exist in two cell-bound forms (TNF-Rs) type 1 (TNF-R1, p55) and 2 (TNF-R1, p75), as well as soluble forms (sTNF-Rs), sTNF-R1 and sTNF-R2, respectively. Binding to TNF-Rs, TNF-α activates the constitutive shedding processes of soluble receptors, that play an important role in regulating its in vivo activity [4–7]. High sTNF-Rs serum/plasma concentrations were found in infections and inflammatory diseases [8, 9]. Dysregulation of the TNF-α pathway is a key feature and pathogenic factor in various diseases, including sepsis, cancers, as well as autoimmune and inflammatory diseases [10]. Fas ligand (FasL CD95L), a membrane molecule, belonging to the TNF family, interacts with Fas (CD95/Apo-1)—a cell surface receptor that is regulated by their soluble forms sFasL and sFas, respectively [11, 12]. The Fas/FasL system is known as one of the most important apoptosis signaling pathways, but its non-apoptotic function is not yet fully understood. The Fas/FasL system is involved in the maintenance of immune homeostasis and its dysfunction is associated with immunological and inflammatory processes [11–14]. The role of TNF family molecules involved in inflammation and apoptosis in CSU has not yet been fully understood [15, 16]. Therefore, it seems important to determine circulating concentrations of TNF-α and their soluble receptors, as well as the sFas/sFasL system in individuals with CSU.

## Materials and methods

The study involved 17 men and 41 women with active CSU [median age 39 (range 21–45 years)]. The control group consisted of 22 healthy people who were matched for age and sex with CSU subjects; recruited from the hospital staff [17].

The subjects were part of a study regarding inflammatory reaction in chronic urticaria, the protocol and clinical examination of which have been described elsewhere [17, 18]. CSU subjects were not on active treatments. Antihistamines have been discontinued for at least 4 days. Treatment with cyclosporine and glucocorticoids has not been used for the period of at least 2 months preceding the study. Cyclosporine was discontinued only in the case of ineffectiveness or adverse reactions. In addition, none of these subjects had previous omalizumab treatment. Subjects were included in the study if the symptoms lasted longer than 6 months. The mean CSU duration was 30.5 months (range 7–62 months).

All subjects gave written informed consent and the Bioethics Committee of the Medical University of Silesia approved the study.

The concentrations of studied parameters in serum samples were measured by the enzyme-linked immunosorbent assay (ELISA) using commercially available kits according to manufacturer’s detailed instructions: for Tumor Necrosis Factor alpha (TNF-α)—Quantikine ELISA Human TNF-α kit, for Fas—Quantikine ELISA Human Fas/TNFRSF6 kit, for Fas Ligand (FasL)—Quantikine ELISA Human Fas Ligand/TNFSF6 kit, for soluble TNF receptor I (sTNF RI)—Quantikine ELISA Human sTNF RI/TNFRSF1A kit and for soluble TNF receptor II (sTNF RII)—Quantikine ELISA Human TNF RII/TNFRSF1B kit (all kits from R&D Systems, MN, USA). The coefficients of variance for intra-assay and inter-assay were below 8% and 10%, respectively.

The obtained results were presented using basic parameters of descriptive statistics. Normal distribution of data was verified using Shapiro–Wilk’s test.

A comparison of two independent groups (CSU subjects as compared to the control group) was performed using the non-parametric U Mann–Whitney test. Independent data between the three groups (moderate-severe CSU vs. mild CSU vs. controls) were compared using the ANOVA rang Kruskal–Wallis’ test and appropriate post hoc tests. The Spearman’s rank test was used for correlations. The value of p < 0.05 was considered statistically significant. The calculations were performed with STATISTICA for Windows 10.0 software (StatSoft, Cracow, Poland).

## Results

### Serum concentrations of TNF-α and its receptors

**Fig. 1:**
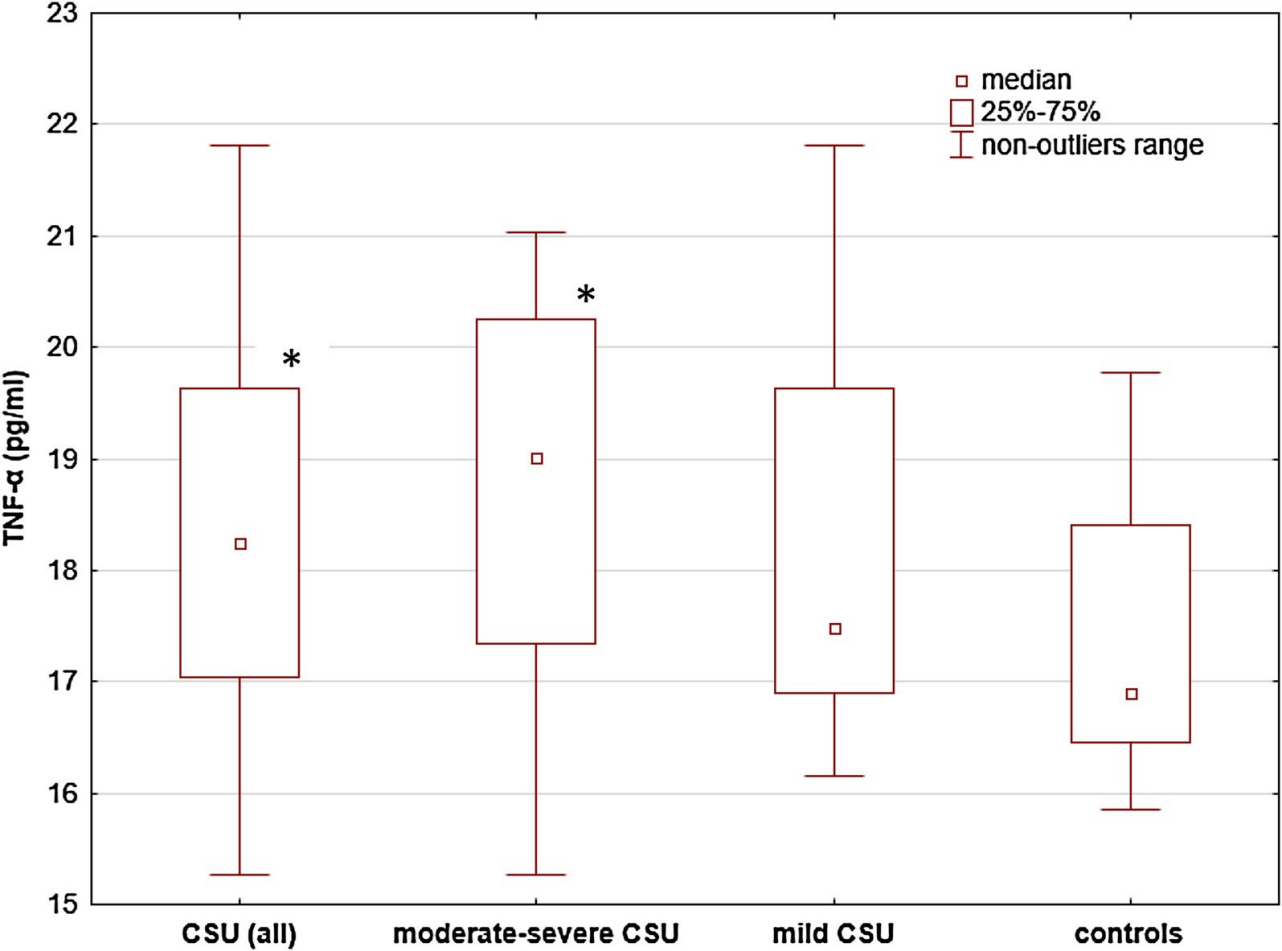
TNF-α concentrations in subjects with chronic spontaneous urticaria (CSU) and the healthy subject. TNF-α concentrations were significantly higher in CSU subjects and moderate-to-severe CSU than in the controls (p < 0.05). There were no significant differences between mild and moderate-severe CSU, and between mild CSU and the controls

TNF-α concentrations were significantly higher in CSU subjects and moderate-to-severe CSU than in the controls [median and IQR 18.25 (17.04–19.62) and 19.01 (17.34–20.24) vs 16.89 (16.45–18.40) pg/ml; *p* < 0.05] (Fig. 1), while there were no significant differences in TNF-α concentrations between subjects with mild [median and IQR 17.49 (16.90–19.62) pg/ml] and moderate-to-severe CSU [median and IQR 19.01 (17.34–20.24) pg/ml] (Fig. 1). Similarly, there were no significant differences in TNF-α concentrations between mild CSU subjects and the controls (Fig. 1).

sTNF-R1 and sTNF-R2 concentrations [median and IQR 1.28 (1.03–1.56) and 2.79 (2.09–3.66) ng/ml] were significantly higher in CSU subjects as compared with the healthy subjects [median and IQR 0.73 (0.67–0.86) and 1.45 (1.26–1.77) ng/ml, respectively; *p* < 0.00001].

**Fig. 2:**
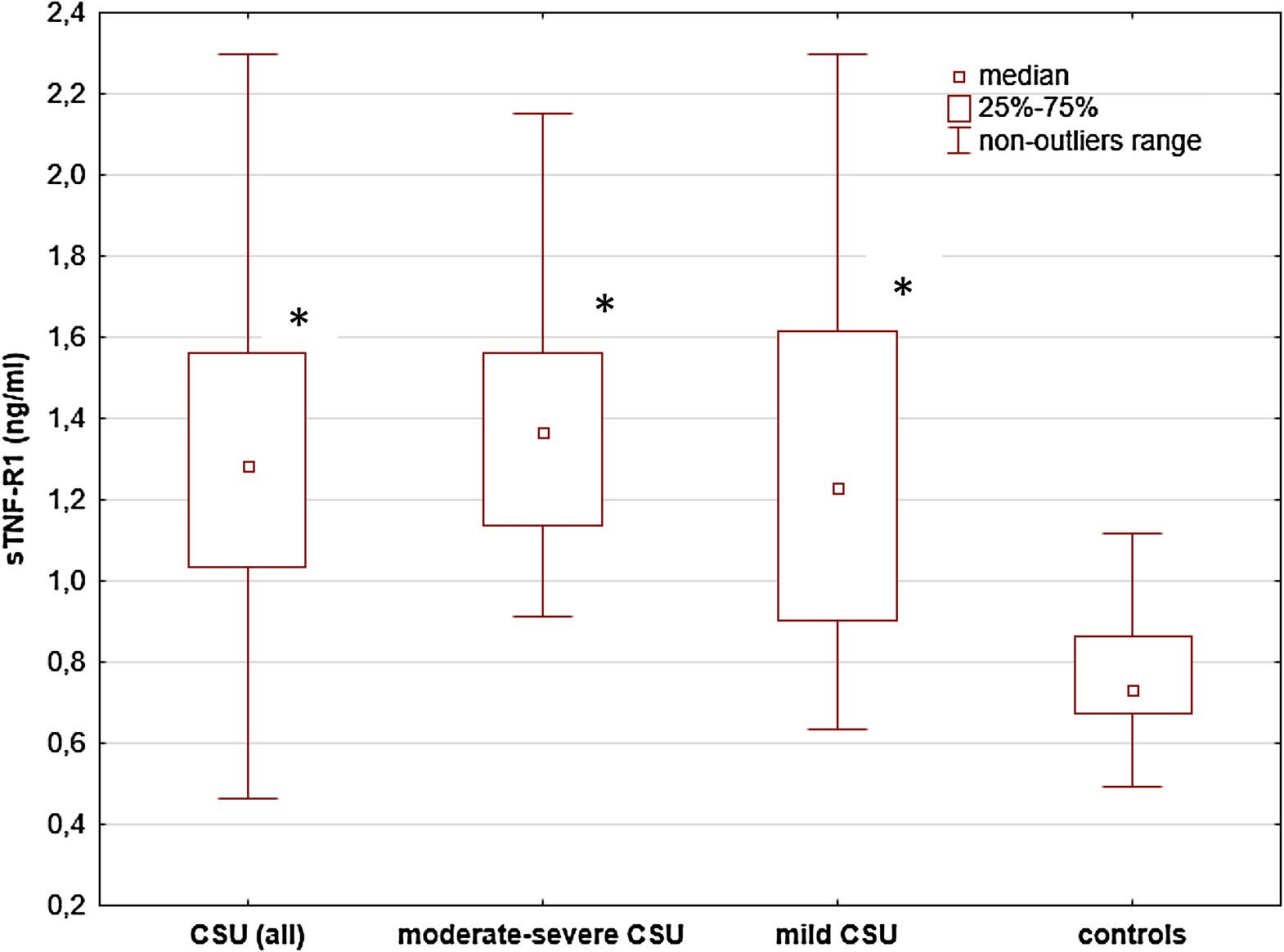
sTNF-R1 concentrations in serum of subjects with chronic spontaneous urticaria (CSU) and the healthy subject. sTNF-R1 concentrations were significantly higher in all CSU and moderate-severe CSU subjects vs. the controls (*p* < 0.00001). The concentrations were also significantly higher in mild CSU vs. the controls (p < 0.0001), but not in moderate-severe CSU vs. mild CSU (p > 0.05)

**Fig. 3:**
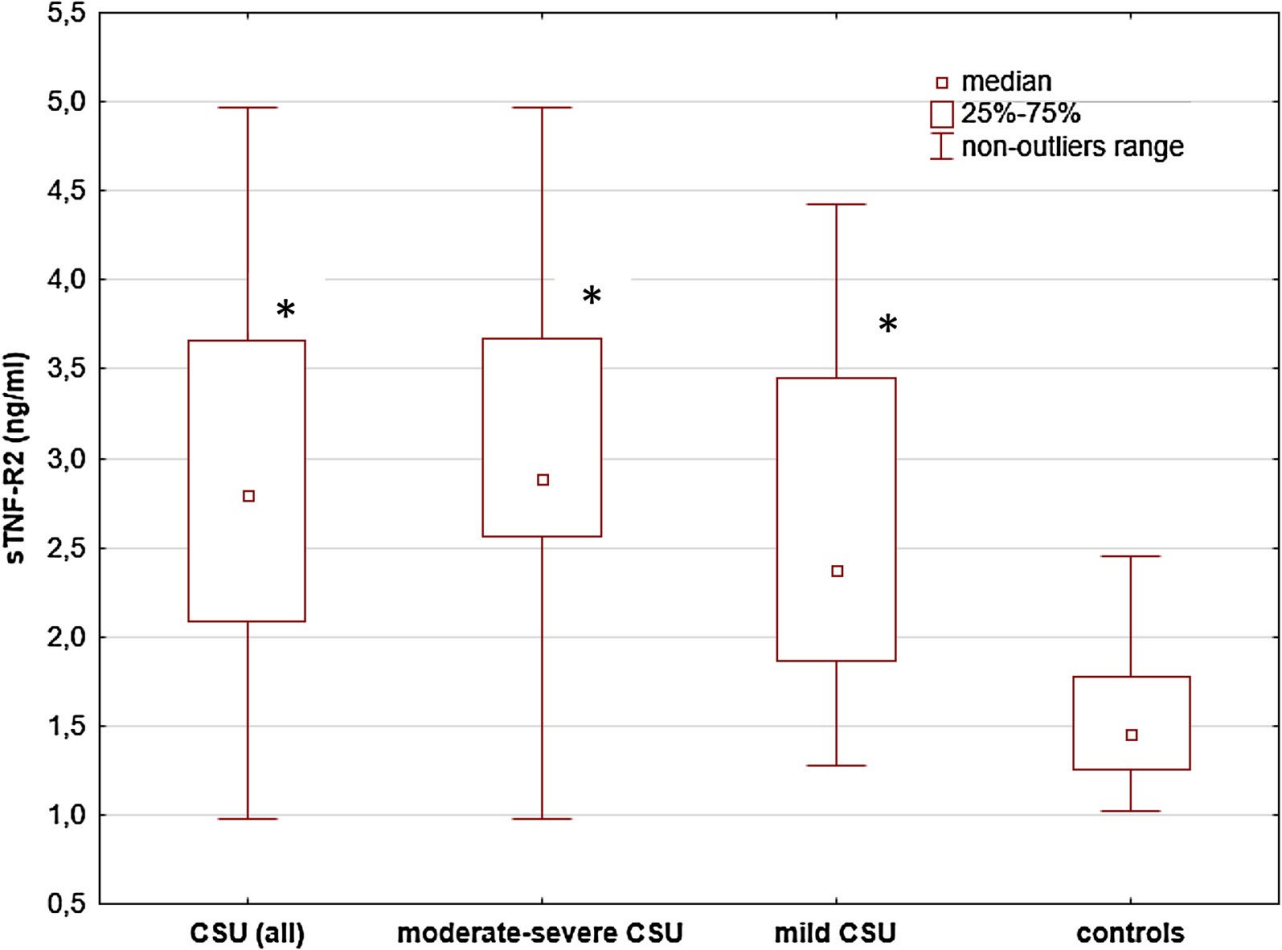
sTNF-R2 concentrations in serum of subjects with chronic spontaneous urticaria (CSU) and the healthy subject. sTNF-R2 concentrations were significantly higher in CSU and moderate-severe CSU subjects vs. the controls (*p* < 0.00001). The concentrations were also significantly higher in mild CSU vs. the controls (p < 0.0001), but not in moderate-severe CSU vs. mild CSU (p > 0.05)

Serum concentrations of sTNF-R1 and sTNF-R2 were significantly higher in moderate-to-severe CSU vs. the controls [median and IQR 1.37 (1.14–1.56) and 2.89 (2.56–3.67) vs. 0.73 (0.67–0.86) and 1.45 (1.26–1.77) ng/ml, respectively; p < 0.00001], but not vs. mild CSU subjects [1.23 (0.9–1.62) and 2.38 (1.87–3.45) ng/ml, p > 0.05] (Figs. 2, 3).

Similarly, sTNF-R1 and sTNF-R2 concentrations were also significantly higher in mild CSU than in the healthy subjects [median and IQR 1.23 (0.9–1.62) and 2.38 (1.87–3.45) vs. 0.73 (0.67–0.86) and 1.45 (1.26–1.77) ng/ml, respectively; p < 0.0001] (Figs. 2, 3).

### Serum concentrations of sFas and sFasL

No significant differences were observed in sFas and sFasL concentrations between all CSU, mild, moderate-to-severe CSU subjects and the controls [median and IQR 15.96 (12.29–21.61) and 87.59 (54.64–106.6), 17.09 (14.33–24.15) and 76.78 (45.23–97.03), 13.79 (10.20–21.50) and 102.03 (67.76–114.88) vs. 12.78 (10.3–19.26) and 75.71 (53.76–91.58) ng/ml and pg/ml, respectively; p > 0.05).

### Serum C reactive protein (CRP) concentrations

CRP concentrations were significantly higher in all CSU subjects, mild, and moderate-severe CSU as compared to the controls as well as between the groups (data not shown, published earlier) [17].

### Correlations

**Fig. 4:**
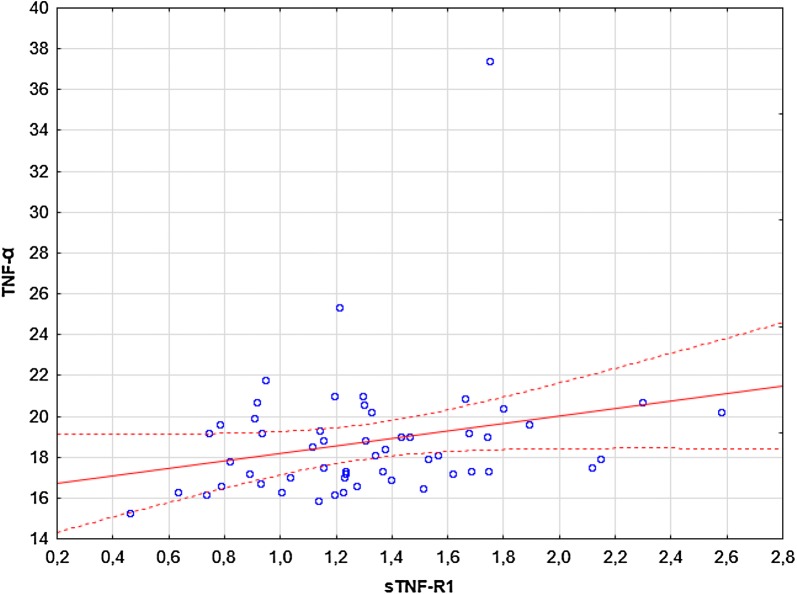
The correlation between TNF-α and sTNF-R1 concentrations in CSU subjects. The blue data points represent the values of the variables tested for individual observations. Solid red lines representing regression lines and broken red lines represent 95% confidence intervals

**Fig. 5:**
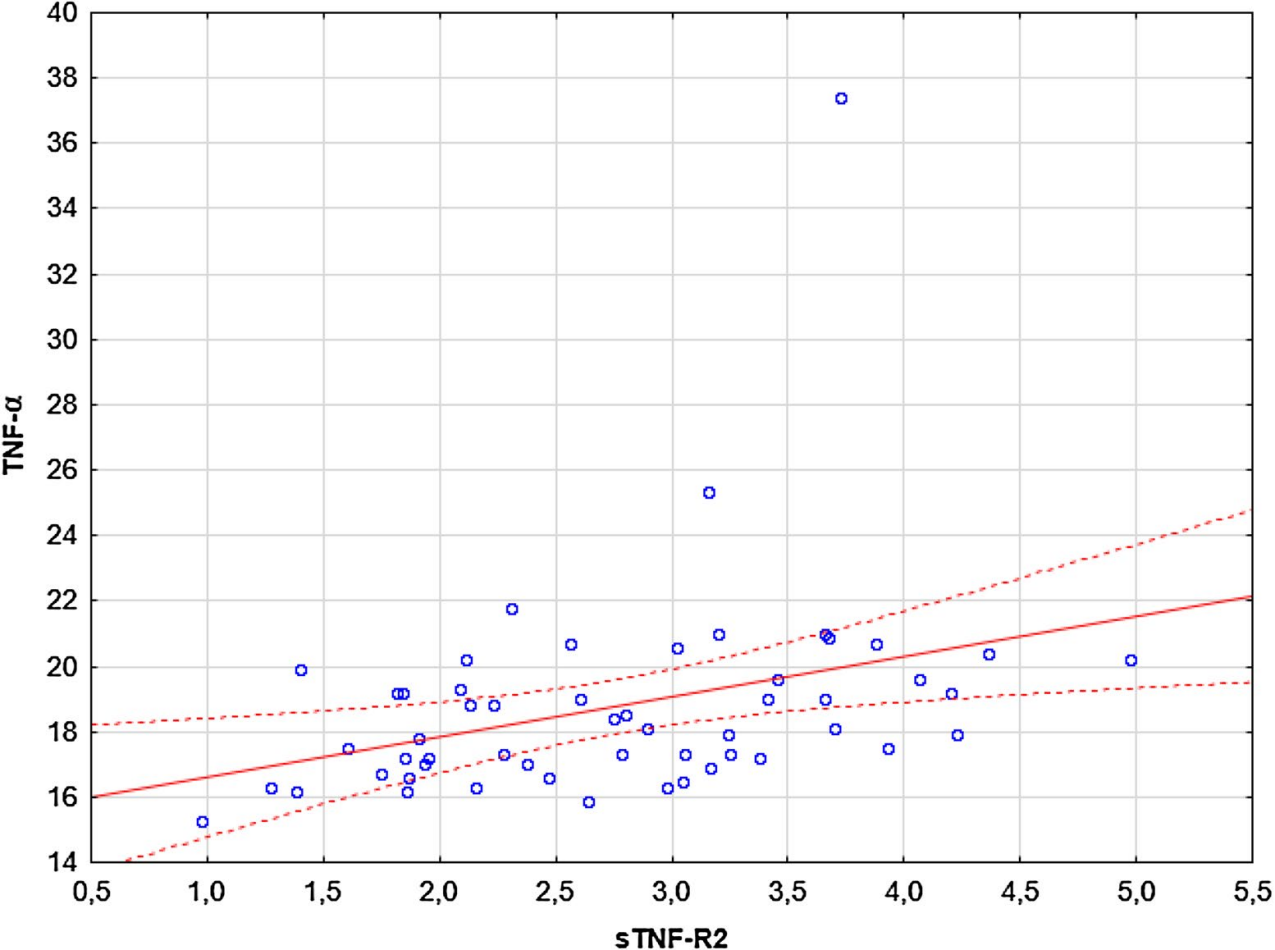
The correlation between TNF-α and sTNF-R2 concentrations in CSU subjects. The blue data points represent the values of the variables tested for individual observations. Solid red lines representing regression lines and broken red lines represent 95% confidence intervals

**Fig. 6:**
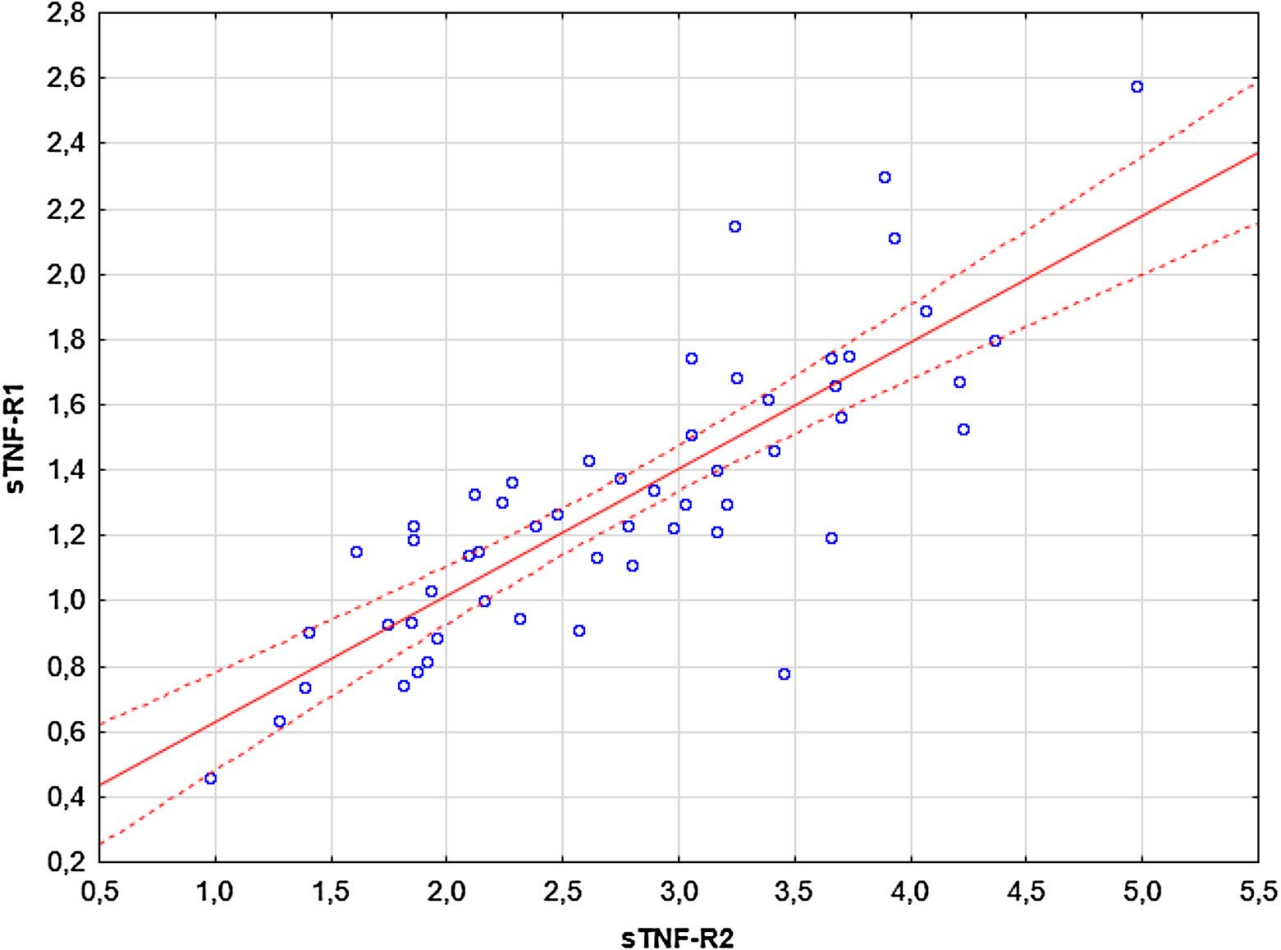
The correlation between sTNF-R1 and sTNF-R2 concentrations in CSU subjects. The blue data points represent the values of the variables tested for individual observations. Solid red lines representing regression lines and broken red lines represent 95% confidence intervals

Significant correlations were observed between: (i) TNF-α and sTNF-R1 (R = 0.28, p = 0.036; Fig. 4), (ii) TNF-α and sTNF-R2 (R = 0.44, p = 0.00078; Fig. 5), (iii) sTNF-R1 and sTNF-R2 (R = 0.79, p = 0.00000; Fig. 6). No significant correlations were observed between: (i) CRP and TNF-α, sTNF-R1, sTNF-R2, sFas, sFasL, (ii) sFas and sFasL, (iii) sFas/sFasL and TNF-α/sTNF-R1/sTNF-R2 (data not shown). There were no significant correlations between UAS4 and TNF-α, sTNF-R1, sTNF-R2, sFas, sFasL (R = 0.26, p = 0.052; R = 0.22, p = 0.09; R = 0.2, p = 0.13; R = -0.16, p = 0.22; R = 0.14, p = 0.29, respectively).

## Discussion

In patients with different types of urticaria, increased TNF-α activity was detected in skin biopsies [19]. In addition, it has been suggested that TNF-α promoter polymorphisms may induce upregulation of TNF-α [20]. In our study, serum TNF-α concentrations were significantly higher in all CSU and moderate-to-severe CSU than in the control group, but not in mild CSU. These results confirm previous observations and suggest the role of the TNF-α system in the pathogenesis of CSU [15, 16].

TNF-α is one of four key cytokines (IL-1, IL-6 and IL-8) involved in the activation of the acute phase response. We have previously found a significant correlation between increased IL-6, IL-8 and CRP concentrations in CSU subjects [3, 21]. In the present study, there was no significant correlation between TNF-α and CRP, which may indicate that TNF-α does not play a significant role in the production of CRP in CSU.

However, circulatory TNF-α is unstable, therefore to get a more precise insight into TNF-α/receptors system activity, both TNF-α and its soluble receptors, which maintain a more stable form should be measured. The soluble TNF-α receptors are more useful for monitoring the activity of the TNF-α system activity [7, 22]. The importance of soluble TNF-α receptors in CSU is unknown.

In our study, the concentrations of sTNF-R1 and sTNF-R2 were significantly higher in all CSU subjects, moderate-to-severe CSU, and mild CSU as compared with the healthy group.

However, no significant differences in these values were observed between subjects with moderate-to-severe and mild CSU. There was no correlation between TNF-α sTNF-R1, sTNF-R2 and UAS in CSU.

It has been suggested that circulating concentrations of sTNF-Rs may reflect the state of activation of the TNF-α/receptors system [7, 22]. Therefore, our results may indicate that CSU is associated with increased activity of the TNF-α/receptors system. In addition increased circulating concentrations of sTNF-R1 and sTNF-R1 may reflect the systemic activation of an immune-inflammatory response in CSU, rather than their increased release form the urticarial lesion into circulation. It has been demonstrated that circulating levels of sTNF-R1 and sTNF-R2 are strongly correlated with the levels of such receptors spontaneously released by isolated peripheral blood T cells in vitro in rheumatoid arthritis [23].

Binding of TNF-α to TNF-Rs activates the constitutive shedding process of soluble receptors, which plays an important role in regulating its in vivo activity [6, 22]. sTNF-Rs may modulate TNF-α activity likely in two different manners depending on their concentrations [24].

In the negative feedback mechanism, high concentrations of TNF-R act as inhibitors/antagonists of TNF-α activity, preventing its binding to membrane-bound TNF-R, maintaining homeostasis and protecting against overwhelming immunological-inflammatory activation [6, 22, 25].

On the other hand, it has been shown that sTNF-Rs at low/intermediate concentrations may preserve and enhance some of the effects of TNF-α by stabilizing its structure in complexes and prolonging its half-life [22, 25].

We observed significant correlations between concentrations of TNF-α and its receptors as well as, sTNF-R1 and sTNF-R2 in CSU.

The open question is whether the increased release of sTNF-Rs in CSU is sufficient to neutralize TNF-α activity to prevent over-response of the TNF/receptors system, systemically and locally at sites of inflammation. One the one hand, we speculate that the higher release of TNF-α in CSU stimulates shedding of sTNF-Rs into the circulation, probably leading to neutralization of TNF-α activity and attenuation/limitation of the inflammatory response, similarly as suggested in other immune-inflammatory diseases [22, 26]. However, it has also been found that their concentration may be insufficient to neutralize the excessive TNF-α response in some chronic inflammatory diseases [9]. Therefore, insufficiently raised circulating concentration of sTNR-Rs might even enhance the effect of TNF-α in urticarial inflammation and systemic inflammatory response by stabilizing its activity and prolonging its function.

However, the source of circulating forms in CSU is still unknown. In contrast to widely expressed TNF-R1, TNF-R2 is expressed typically by immune cells [5, 27], thus an increase in circulating sTNF-R2 concentration may indirectly indicate immune activation in CSU.

The balance between TNF- activity and sTNF-Rs determines the course of immune activation and inflammatory response, and its dysregulation may play a role in susceptibility and resistance to various diseases [4], including sepsis, cancers, as well as autoimmune and inflammatory diseases [10].

In addition, persistent expression of the nonsheddable form of TNF-R1 has been shown to increase susceptibility to hyperreactivity and inflammatory response in mice [4].

The TNF-R1 mutation may result in a decrease in receptor cleavage and a reduction in the shedding of potentially antagonistic sTNR-1 and is associated with certain autoinflammatory syndromes called TNF-R1-associated periodic syndromes [28].

It has been suggested that the Fas/FasL system may have pro-inflammatory activity [29, 30]. The relationship between sFas/sFasL and the activity and severity inflammatory processes has been demonstrated [31, 32].

In contrast to other immune-inflammatory diseases, CSU is not associated with up-regulation of circulating soluble Fas/FasL system. No significant differences were observed in sFas and sFasL concentrations between CSU subjects and the control group.

We only measured circulating concentrations of sFas and sFasL, which may be less important than their local levels at the site of inflammation [33].

Our results do not exclude the importance of the Fas/FasL system and its soluble forms in urticarial inflammation. It seems, however, that concentrations of soluble apoptotic factors in serum/plasma would not be useful indicators of the activity/severity of urticarial inflammation.

TNF-α/receptors and Fas/FasL systems are two key cell death-inducing systems in one apoptotic pathway [11]. Considering the lack of significant changes in circulating sFas/sFasL in CSU subjects, it seems that increased plasma concentrations of TNF-α are related to its pro-inflammatory function rather than the enhanced systemic apoptopic response in CSU subjects.

The use of TNF-alpha inhibitors beyond therapeutic indications may be effective in a significant proportion of patients with refractory chronic urticaria, also previously unsuccessfully treated with omalizumab (anti-IgE) [34, 35].

Sand et al. observed complete or almost complete response in 60% and a partial response in a further 15% of subjects with refractory chronic urticaria during therapy with TNF‐alpha inhibitors (adalimumab or etanercept) [35].

Taking into consideration our and previous results, inhibiting the TNF-α system activity as a therapy for refractory chronic urticaria unresponsive to omalizumab and cyclosporine or associated with unacceptable drug side effects seems to be interesting for further investigations.

Nevertheless, our study has some limitations, therefore we can not prove the existence of any causal relationship. We have not studied the correlation between TNFα/sTNF-Rs and the effectiveness of anti-inflammatory or anti-IgE medications.

## Conclusions

CSU is associated with the activation of the TNF-α signaling pathway, marked by increased circulating concentrations of sTNF-R1 and sTNF-R2, probably as part of the systemic inflammatory response in the disease. TNF-α and its soluble receptors are related to each other in CSU. In contrast, the circulating soluble Fas/FasL system that mainly reflects systemic apoptosis is not upregulated in CSU patients. There is no relationship between TNF-α/sTNF-R1/sTNF-R2 and sFas/sFasL pathways in the disease.

## Abbreviations

CSU: chronic spontaneous urticaria
TNF-α: tumor necrosis factor alpha
TNF-R1: tumor necrosis factor receptor type 1
TNF-R2: tumor necrosis factor receptor type 2
sTNF-Rs: soluble tumor necrosis factor receptors
sTNF-R1: soluble tumor necrosis factor receptor type 1
sTNF-R2: soluble tumor necrosis factor receptor type 2
FasL: Fas ligand
UAS: urticaria activity score
ELISA: enzyme-linked immunosorbent assay
CRP: C reactive protein
